# A Comparative Genomic and Transcriptional Survey Providing Novel Insights into Bone Morphogenetic Protein 2 (*bmp2*) in Fishes

**DOI:** 10.3390/ijms20246137

**Published:** 2019-12-05

**Authors:** Guang Yang, Zhendong Qin, Hongyan Kou, Rishen Liang, Lijuan Zhao, Shoujia Jiang, Li Lin, Kai Zhang

**Affiliations:** 1 Guangdong Provincial Water Environment and Aquatic Products Security Engineering Technology, Research Center, Guangzhou Key Laboratory of Aquatic Animal Diseases and Waterfowl Breeding, Zhongkai University of Agriculture and Engineering, Guangzhou 510225, China; yangguang@m.scnu.edu.cn (G.Y.); qinzhendongsc@163.com (Z.Q.); cheetahliang@126.com (R.L.); zhaolijuan4234@163.com (L.Z.); 2 Guangdong Provincial Key Laboratory for Healthy and Safe Aquaculture, College of Life Science, South China Normal University, Guangzhou 510631, China; 3 Shenzhen Key Lab of Marine Genomics, Guangdong Provincial Key Lab of Molecular Breeding in Marine Economic Animals, BGI Academy of Marine Sciences, BGI Marine, BGI, Shenzhen 518083, China; jiangshoujia@genomics.cn; 4 Division of Life Science, Hong Kong University of Science and Technology, Hong Kong 93117, China

**Keywords:** IBs, *bmp2*, molecular evolution, comparative genomic, transcriptional level

## Abstract

Intermuscular bones (IBs) are only found in the muscles of fish. Bone morphogenetic protein 2 (bmp2) is considered to be the most active single osteogenesis factor. It promotes cell proliferation and differentiation during bone repair, as well as inducing the formation of bones and cartilages in vivo. However, detailed investigations of this family in fish are incredibly limited. Here, we have used a variety of published and unpublished *bmp2* sequences for teleosts and cartilage fish in order to explore and expand our understanding of *bmp2* genes in fish. Our results confirmed that teleost genomes contain two or more *bmp2* genes, and the diversity of *bmp2* genes in vertebrates appears to be as a result of a combination of whole genome duplication (WGD) and gene loss. Differences were also observed in tissue distribution and relative transcription abundance of the *bmp2s* through a transcriptomic analysis. Our data also indicated that *bmp2b* may play an important role in the formation of IBs in teleosts. In addition, protein sequence alignments and 3D structural predictions of *bmp2a* and *bmp2b* supported their similar roles in fishes. To summarize, our existing work provided novel insights into the *bmp2* family genes in fishes through a mixture of comparative genomic and transcriptomic analysis.

## 1. Introduction

Most aquaculture freshwater fish, in particular the cyprinidae species, contain a specific amount of IBs which are hard-boned spicules that can be found in the muscle tissue on both sides of the vertebrae [1]. IBs are only present in the myosepta of lower teleosts, and these are viewed as ossified myoseptal tendons which develop directly from mesenchymal condensation [2]. Previous studies have discovered that the appearance of intermuscular bones (IBs) can enhance the power of herbivorous fish to a certain extent, which means that these fish can better adapt to the environment and evade natural predators [3,4,5]. The presence of IBs influences the farming and processing of teleosts, to some degree, as well as decreasing the economic value and edibleness of the species, due to the fact that they are difficult to remove [6]. While observing the potential value of the eradication of IBs, researchers initiated the research of IBs in fish in the early 1960s [7]. Until now, almost all current research has only focused on the IBs’ morphology in species of fish [8,9,10]. A very small amount of detailed research has been conducted and little information is known in relation to the molecular mechanism for the development of IBs.

Bone morphogenetic proteins (bmps) are active proteins which were extracted from adult bone tissue by Urist in 1965. Apart from bmp1, other bmps are members of the changing growth factor-beta (TGF-β) superfamily, which promote the formation of cartilage and bone tissue [11,12]. It was originally believed that bmps only play a role in the formation of bone and cartilage in vertebrates, but now it is believed that bmps play biological roles in various cell types [13]. In addition to inducing the formation of bone and cartilage, bmps are also involved in regulating the development of teeth, kidneys, skin, hair, muscles, hematopoiesis, and neurons, as well as maintaining iron metabolism and vascular homeostasis in the body [14,15]. Bmp2 is considered to be the most active single factor of osteogenesis [16]. Bmp2 has been widely studied in the bmp family since 1988, and it has played an important role in bone induction, and become a therapeutic agent for the restoration of bones and teeth [17,18]. Most data indicate that bmp2 is associated with the initial growth of various organs and tissues of vertebrates, and that it is also involved in the early development of the skeletal system and the formation of various organs [19,20]. Most current research into the *bmp2* gene focuses on its role in bone fragmentation and repair. The role of the *bmp2* gene is still unclear in relation to the formation of IBs in teleosts.

In recent years, the study of bmp2 has been extended to fish models in bone biology, in particular the zebrafish (*Danio rerio*). Mowbray defined the mRNA expression patterns in the ears of developing zebrafish as bmp2b, and provided supporting evidence that bmps play a vital role in a vertebrate’s ear development [21]. Spatiotemporal expression patterns of bmp2 in zebrafish and Senegalese sole (*Solea senegalensis*) revealed that all isoforms are stated in calcified tissues, but at a variety of levels [22,23]. Also, bmp2a and bmp2b have been recognized as being part of zebrafish fin regeneration or development [24,25]. Zebrafish swirl mutant which is triggered by alterations in the *bmp2b* gene [26] indicates that bmp2b is vital during the early dorsoventral patterning. The studies of components of *bmp2* genes in other species of fish relating to bone biology are inadequate, and most studies relate to the copy number of *bmp2* genes. The *bmp2* family is present in different members of different species. Most tetrapods harbor a single *bmp2* gene, while two *bmp2* genes are reportedly presented in teleosts [27]. Previous studies have shown that the *bmp2b* gene has the highest expression level in the muscles of common carp (*Cyprinus carpio*) [28]. Furthermore, the *bmp2a* gene is highly expressed in calcified bone tissue of the gilthead seabream (*Sparus aurata*) [29] and in the ovaries of African clawed frogs (*Xenopus laevis*) [30].

Although expression patterns of *bmp2* genes have been reported in a few boned fishes, a comprehensive and comparative genomic and transcriptomic survey of these family genes in various species of fish has not yet been undertaken. In this study, we extracted fish *bmp2* genes (and their encoding sequences) from several published genome sequences, in order to allow us to investigate the presence or absence of species-specific *bmp2* isotypes and their sequence differences throughout various species. Comprehensive analysis was conducted for *bmp2* genes in fish, including the analysis of molecular structures, gene copy numbers, and phylogenetic relationships. Furthermore, the tissue distribution of these genes was determined. Our results lay a solid foundation for further functional studies of *bmp2* genes in fish.

## 2. Results

### 2.1. Variation of bmp2 Copy Number in Different Fish Species for a Phylogenetic Analysis

This study analyzed the bmp2a and bmp2b sequences (including gene sequence information and protein sequence information) of 37 different vertebrates, including 28 species of fish. Table 1 displays information of the *bmp2* gene copy number which was obtained from the genome of 28 species of fish and one species of amphibian using bioinformatics. Table S1 shows the publicly-reported sequence information of *bmp2a* and *bmp2b* from 16 species of vertebrate (including eight species of fish and eight species of mammal).

We performed gene copy number analysis of the *bmp2* gene in fish genomes. Based on the results of our analysis, the copy number of the *bmp2* genes from different species has a certain variation. There is only one *bmp2* gene in the tetrapod genome (*Homo sapiens, Mus musculus, Ornithorhynchus anatinus, and Pan troglodytes*), whereas there are two or more in the teleost genome. There are two *bmp2* genes (*bmp2a* and *bmp2b*) in the genome of diploid teleosts, such as Nile tilapia (*Oreochromis niloticus*) and bluntnose black bream (*Megalobrama amblycephala*), yet there are exceptions as there are four *bmp2* genes in the Atlantic salmon (*Salmo salar*) genome (two *bmp2a* and two *bmp2b*). We discovered multiple copies in tetraploid teleosts, including surface-dwelling golden-line fish (*Sinocyclocheilus grahami*, Sg), semi-cave-dwelling golden-line fish (*Sinocyclocheilus rhinocerous*, Sr), and cave-restricted golden-line fish (*Sinocyclocheilus anshuiensis*, Sa). Golden-line fish have three or four *bmp2* copies. In addition, we analyzed the copy number of *bmp2* genes in some ancient fish and discovered that only one copy of the *bmp2b* gene was present in the spotted gar (*Lepisosteus oculatus*) and golden arowana (*Scleropages formosus*).

In addition, we found that both diploid and polyploid fishes contain at least one copy of the *bmp2b* gene. Interestingly, we discovered that some marine fish species lost their *bmp2a* genes, including the large yellow croaker (*Larimichthys crocea*) and black porgy (*Acanthopagrus schlegelii*). This could have been due to gene loss during evolution or the inability to excavate the *bmp2b* gene from these species due to genome assembly problems.

**Figure 1:**
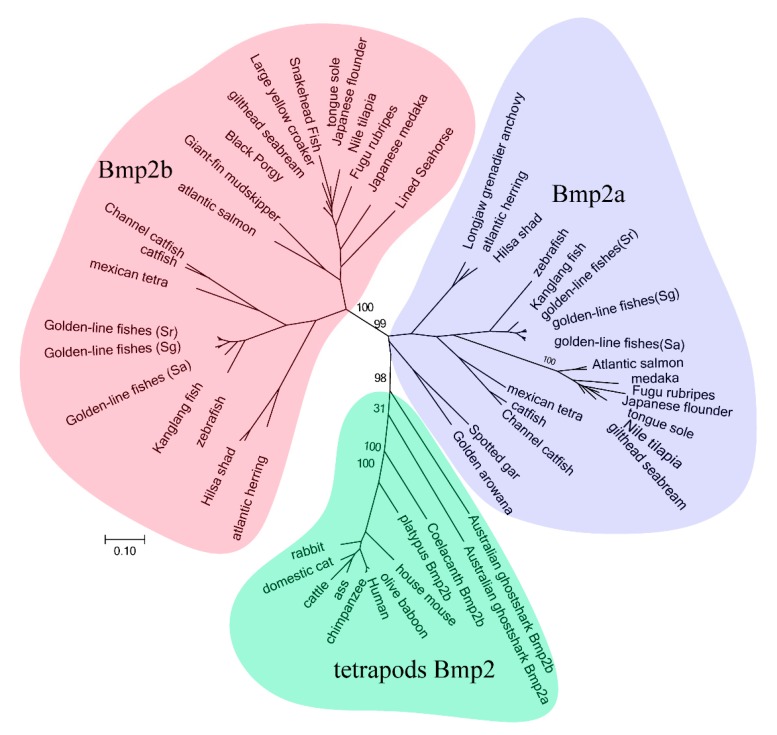
Clustering of *bmp2* genes. The phylogenetic analysis was performed by RAxML8.0.17. Numbers on branches are bootstrap values. Scale bar indicates the rate of amino acid substitution per residue. Although the MEGA tree is not shown here, it possesses the same tree topology. Different bmp2 subfamilies are displayed with various colors: blue, teleost bmp2a; green, tetrapod bmp2; and pink, teleost bmp2b.

We constructed a phylogenetic tree, using all the acquired protein sequences of the *bmp2* genes, as displayed in Figure 1. Our results showed that all genes were clustered into three main branches. The *bmp2a* genes of all fish and the *bmp2* genes of tetrapods clustered into a large branch. However, this large branch formed two small branches, namely the *bmp2a* gene branch of the teleosts and the *bmp2* genes branch of the tetrapods. All *bmp2b* genes of the teleosts were clustered into a branch. The phylogenetic tree of the *bmp2* gene family was constructed using phylogenetic analysis in order to determine the specific subtype and evolutionary status of the *bmp2* genes of certain species.

### 2.2. Synteny Data

**Figure 2:**
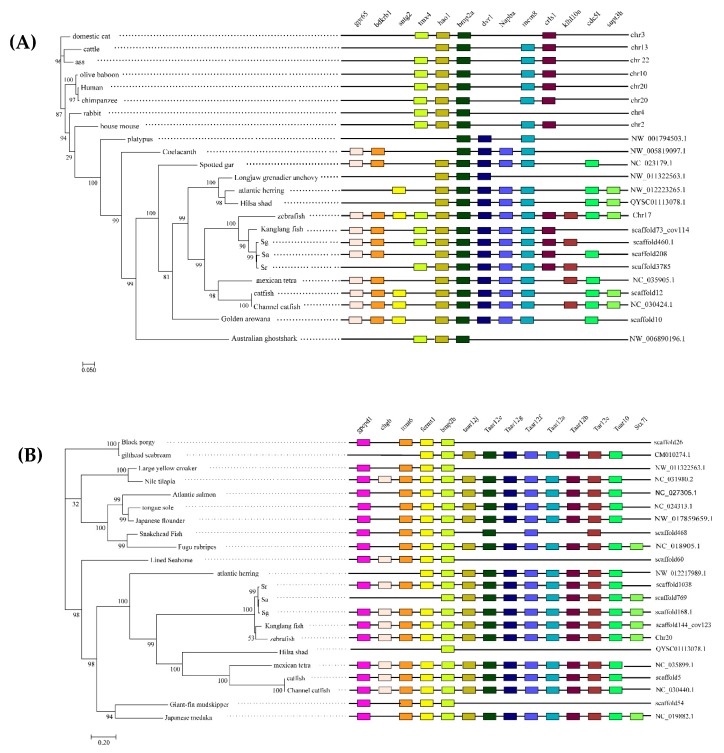
Phylogenetic trees and synteny of *bmp2a* (**A**) and *bmp2b* (**B**). These maximum likelihood (ML) trees (on the left-hand side of each figure) were constructed by FastTree v2.1.7. Bootstrap values are shown on branches. The right-hand figures are synteny of the two *bmp2* genes.

We used the zebrafish *bmp2a* and *bmp2b* gene sequences as reference points, and then selected multiple upstream and downstream genes. The protein sequences of these genes were downloaded and compared to the genomes of other species in order to find the two genes on the genomes and the location of the upstream and downstream genes. The specific results are shown in the right-hand side of Figure 2. From the results of the collinear analysis, it can be noted that even though certain species have some gene loss, the distribution of *bmp2a* and *bmp2b* genes in the teleost genome is generally conserved. For the most part, the *hao1* gene and the *tmx4* gene are present upstream of the *bmp2a* gene in the genome of teleosts, and the *drv1* gene, the *napba* gene, the *mcm8* gene, and the *crlsl* gene are present downstream of the *bmp2a* gene in the teleost genome. The upstream and downstream genes of the *bmp2b* gene are highly conserved in the teleost genome. The *Gpcpd1* gene, *trmt6* gene, and *fermt1* gene exist upstream of the *bmp2b* gene in the teleost genome. *TAAR12j* genes, *TAAR12e* genes, *TAAR12g* genes, *TAAR12f* genes, *TAAR12a* genes, *TAAR12b* genes, *TAAR12c* genes, and *TAAR10* gene exist in the downstream of *bmp2b* genes in the teleost genome. We found that the distribution of the *bmp2* genes on the mammalian genome is different to the distribution of the *bmp2a* genes in teleosts. Significant gene loss was discovered in the upstream and downstream of the mammalian *bmp2* gene. A significant amount of gene loss was discovered upstream and downstream of the *bmp2a* and *bmp2b* genes of the Australian ghostshark (*Callorhinchus milii*). The collinear analysis also confirmed that the *bmp2a* and *bmp2b* genes we extracted were reliable.

### 2.3. Sequence Alignment and Three-Dimensional (3D) Structure Prediction of Zebrafish bmp2a Gene and bmp2b Gene

We predicted that the domains of the zebrafish *bmp2a* gene and *bmp2b* gene consisted of three parts, both of which contained the TGFb_propeptide domain and the TGFB domain. Bmp2a also contains a low complexity region, and bmp2b contains a signal peptide. TGFb_propeptide represents the propeptide region of TGF-β forming Latency associated peptide (LAP). TGF-β is concealed as a potential complex, and is comprised of two parts: TGF-β dimer and TGF-β binding protein (LTBP). Following translational processing, TGF-β is concealed as a complex consisting of TGF-β dimer and TGF-β binding protein (LTBP) which are non-covalently bound to LAP. The potential TGF-β can be targeted to the extracellular matrix by LTBP. LAP dimers typically bind to LTBP with disulfide bonds. TGFB belongs to the transmuting growth factor-beta (TGF-β) family. TGFB is a multi-functional peptide which controls the proliferation, differentiation, and other functions of several cell types.

**Figure 3:**
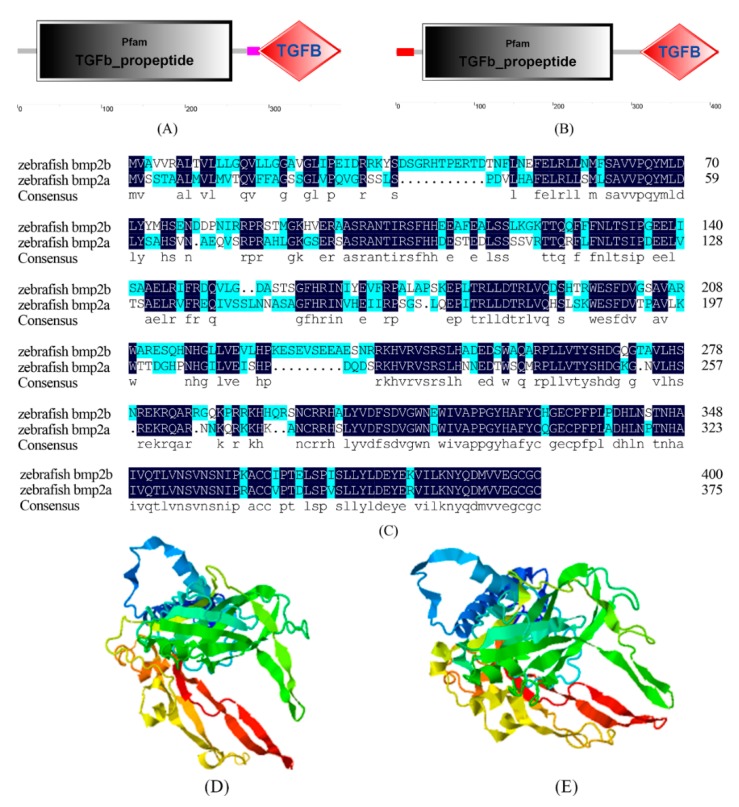
Alignment of bmp2 protein sequences of zebrafish. (**A**) The domain of zebrafish bmp2a, (**B**) the domain of zebrafish bmp2b, (**C**) alignment of bmp2a and bmp2b protein sequences of zebrafish, (**D**) predicted 3D models of bmp2a and bmp2b of zebrafish, and (**E**) predicted 3D models of bmp2b of zebrafish.

Both the *bmp2a* gene and *bmp2b* gene of the zebrafish contain a common receptor protein with an aserine/threonine kinase activation site in the spatial structure (see Figure 3). The serine/threonine kinase activation site residues on bmp2a are located at amino acid positions (286, 288, 291, 293, 310, 313, 315, 317, 351, 353, 356, 378, and 383). The serine/threonine kinase activation site residues on bmp2b are located at amino acid positions (311, 313, 316, 318, 335, 338, 340, 342, 376, 378, 381, 384, 403, and 408). Both bmp2a and bmp2b in zebrafish have a receptor protein with the serine/threonine kinase activation site, which suggests that this site may be significant for maintaining the function of the zebrafish *bmp2* gene family.

### 2.4. Structural Analysis of bmp2a and bmp2b

**Figure 4:**
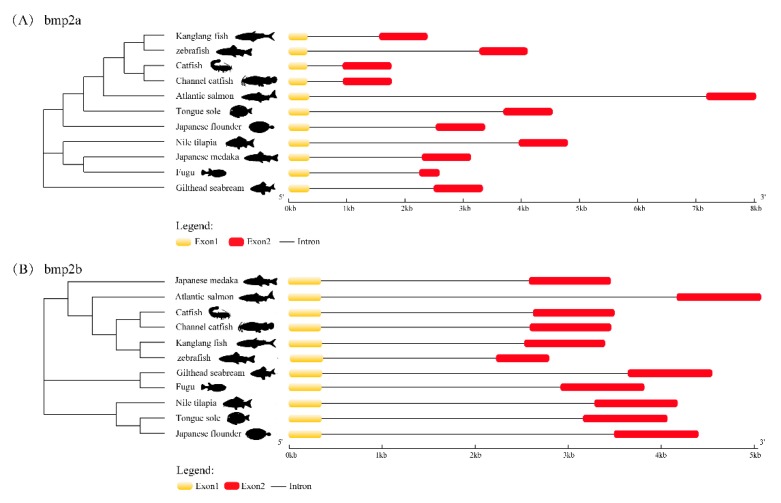
Exon–intron structures of *bmp2a* (**A**) and *bmp2b* (**B**). Boxes in red and yellow represent the exons, while gray lines represent the introns.

Through the structural analysis of the *bmp2a* gene and *bmp2b* gene (see Figure 4), it was discovered that both the *bmp2a* gene and *bmp2b* gene of teleosts contain two exons, and the exons and introns are arranged in a very conserved gene. The model is consistent, and this suggests that *bmp2a* and *bmp2b* genes could have acquainted functions.

**Figure 5:**
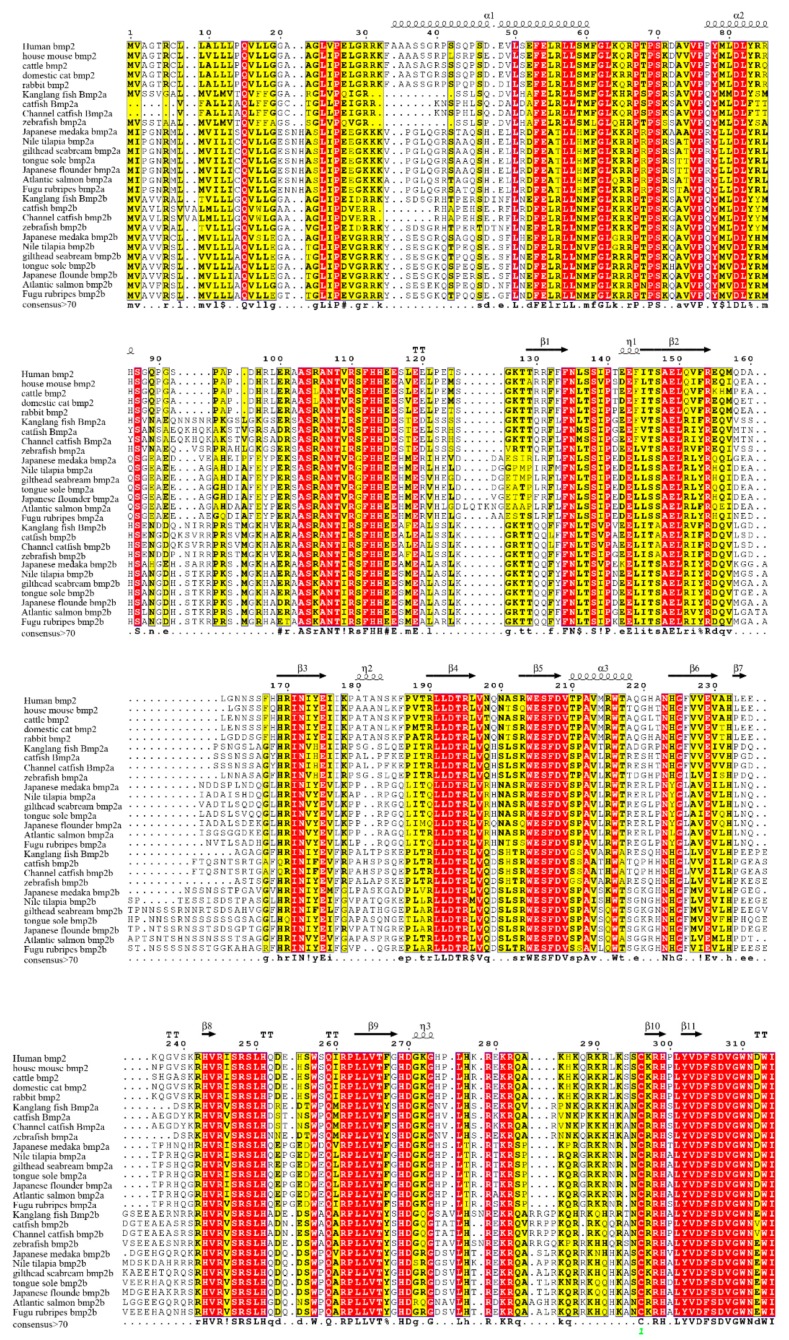

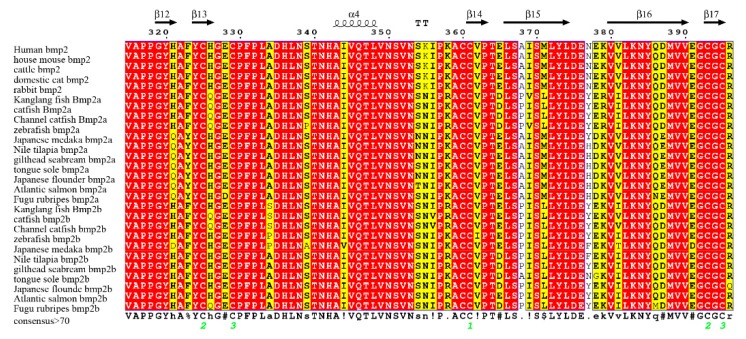
Alignment of bmp2 protein sequences. These sequences were aligned with human bmp2 by multiple sequence alignment based on fast Fourier transform (MAFFT) and colorized using TEXshade. The secondary structural elements, alpha helix (α) and beta strand (β), are marked. The color code for the conservation track ranges from red (the most conserved) to yellow (the least conserved) as per TEXshade.

We downloaded the three-dimensional structure information of human bmp2 protein from the Protein Data Bank (PDB) database as a template for our analysis. The sequence alignment results and three-dimensional structure analysis results are displayed in Figure 5. We focused on amino acid sites which were thoroughly linked to the function and structure of bmp2 proteins. It was discovered that certain amino acids around the 2HP ligand binding site were conserved in *bmp2a* and *bmp2b* genes. For example, the two proteins of all species were isoleucine (Ile) residues, proline (Val) residues, alanine (Ala) residues, and proline (Pro) residues at positions 304, 305, 306, and 307 (relative to the bmp2a protein of zebrafish, see Figure 5). These amino acid residues can maintain the stability of the 2HP ligand. Bmp2a protein and bmp2b protein were also conserved at the DIO ligand binding site. For example, two proteins residues are tryptophan (Trp) residues, cysteine (Cys) residues, and tyrosine (Tyr) residues at positions 301, 303, and 363. However, it was very different at positions 37, 60, and 124. The sites are proline (Pro) at position 37 in most mammals, while the bmp2a is leucine(Leu)in other teleosts, and the bmp2b is phenylalanine(Phe) in most teleosts. The sites are aspartic acid (Asp) at position 60 in most mammals, while the bmp2a is serine (Ser) in other teleosts, and the bmp2b is glutamine (Glu) in most teleosts. At position 124, mammalian bmp2 and teleost bmp2a are arginine (Arg), while teleost bmp2b is glutamine (Glu). These key sites of bmp2 may be involved in the evolution of bmp2 in mammals and teleosts.

### 2.5. Transcriptional Level Studies of Various Tissues of Nile Tilapia and Bluntnose Black Bream

**Figure 6:**
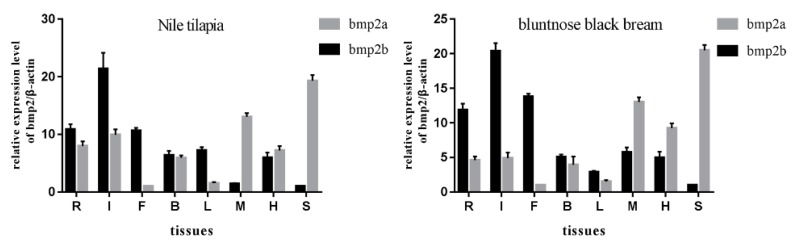
Tissue distribution analysis of *bmp2* of Nile tilapia and Bluntnose black bream were analyzed by quantitative real-time PCR (qRT-PCR). Relative expression levels of *bmp2a* and *bmp2b* in the gill, muscle, stomach, heart, brain, and hemolymph, with β-actin as an internal control. Letters R, I, F, B, L, M, H, S stand for rib, intermuscular bones (IB), fin, brain, liver, muscle, heart, and spleen, respectively.

We chose two representative teleosts: the Nile tilapia and the bluntnose black bream. Eight tissues, including IBs, spleen, heart, muscle, liver, brain, and fin were selected as research objects for the Nile tilapia and bluntnose black bream. Using β-actin as an internal reference gene, the expression of *bmp2* genes of Nile tilapia and bluntnose black bream in eight tissues which were ribs, intermuscular spines, fins, brain, liver, muscle, heart and spleen was detected by quantitative real-time PCR (qRT-PCR). The results indicated that the *bmp2* genes were expressed in eight tissues of Nile tilapia and bluntnose black bream, but the expression levels were all different (see Figure 6). *Bmp2a* had the highest expression in the spleen, followed by IBs, ribs and fins, and muscle, brain, and liver, with the lowest expression being in the heart of the bluntnose black bream. *Bmp2b* was highly expressed in the IBs, ribs, and fins, and the expression in the liver, brain, and muscle was second. The expression in the heart and spleen was the lowest in the bluntnose black bream. The expression of the *bmp2* genes in various tissues of the Nile tilapia was similar to that in the bluntnose black bream. *Bmp2a* had the highest expression in muscle, IBs, rib, and fin, followed by spleen and brain, with the lowest expression in the liver and heart of the Nile tilapia. *Bmp2b* was highly expressed in the muscle, liver, and the spleen, and second in ribs, IBs, and fins. The expression was lowest in the brain and heart of the bluntnose black bream.

## 3. Discussion

In this study, we researched several surfaces of *bmp2* genes (with both *bmp2a* and *bmp2b*), and gained novel insights into the structural variations and diversity of *bmp2* in vertebrates from a genomics point of view.

### 3.1. Possible Reasons for Copy Number Variations among Vertebrates

We analyzed variations of copy numbers of the *bmp2a* and *bmp2b* genes in different vertebrates and concluded that the main causes of variations were genome-wide replication and gene deletion. As we are aware from the relevant literature and studies, the general forefather of early vertebrates experienced two rounds of whole genome duplication (WGD) [31,32]. All teleosts are generally considered to have experienced at least three whole genome duplication events [33,34], and some fish even experience a fourth genome duplication [35]. Our results suggested that when tetrapods and teleosts are isolated, tetrapods lose the *bmp2b* gene, and there is therefore a *bmp2a* gene in tetrapods. Two *bmp2* genes (*bmp2a* and *bmp2b*) are generally present in the genome of diploid teleosts, such as the bluntnose black bream. Some fish which have undergone a fourth round of genome duplication, such as the tetraploid golden-line fish and Atlantic salmon, contain two *bmp2a* genes and two *bmp2b* genes in their genomes. The copy number of the *bmp2a* gene in tetraploid fish genomes is not 2:1, compared to the copy number of the *bmp2a* genes contained in the diploid fish genome, which indicates that the *bmp2a* genes have been lost in the genome of tetraploid fish. Therefore, we can conclude that the important factors for copy number variation in the *bmp2a* and *bmp2b* genes are genome-wide duplication and gene deletion. Based on our findings, we projected that the important factors for copy number variations of *bmp2* in vertebrates were caused by a combination of gene loss and WGD. The number of copies between diploid and tetraploid does not always correspond to one-to-two, due to the selective loss of genes.

In addition, our synteny analysis results showed that the *bmp2a* and *bmp2b* genes are restricted in the same species with different chromosomes, whereas all *bmp2a* and *bmp2b* genes, across species share a preserved suite of genes binding them on both sides, although some species may display gene loss. Interestingly, we determined that the synteny genes were not conserved between teleosts and tetrapods, indicating that the *bmp2* family genes experienced rearrangement from teleosts to tetrapods.

### 3.2. Adaptive Evolution of bmp2s in Vertebrates

IBs are common and play a role in muscle support and power transmission in the muscles of lower teleosts [36,37]. The reason for the formation of IBs in lower teleosts could be that they are induced by differentiation from muscle fibroblasts, and muscle mesenchymal cells [38,39]. Additionally, the preference for food among lower teleosts is an important biological phenomenon [40,41,42]. According to our findings, we discovered that herbivorous fish (such as the bluntnose black bream which has more IBs) have more *bmp2* genes than carnivorous fish (such as the snakehead fish which has fewer IBs). Previous studies have reported that the existence of IBs enhances the power of herbivorous fish to a certain extent, which allows these fish to better adapt to their environment and to evade natural predators [3,4,5]. According to our phylogenetic analysis and protein structure comparisons, we observed that some marine fishes, including the large yellow croaker (*Larimichthys crocea*) and black porgy (*Acanthopagrus schlegelii*), only have one *bmp2* gene (*bmp2a*), and this could result in fewer IBs among marine fish.

Through the analysis of the expression levels of *bmp2a* and *bmp2b* in various teleost tissues, we discovered that *bmp2b* is expressed mostly in the IB, ribs, and fins of the Nile tilapia and *bmp2a* is expressed mostly in the spleen, muscle, and heart of the Nile tilapia, which is consistent with previous studies of zebrafish [22,24,43]. With the bluntnose black bream, *bmp2b* were highly expressed in the IB, ribs and fins. Furthermore, a higher expression level of *bmp2a* was observed in the soft tissue of the spleen, muscle, and heart of the bluntnose black bream. This is also consistent with the previous findings [27,44]. Our results suggested that *bmp2b* is preferentially expressed in fishbone-related tissue (such as IB, fins, and ribs), while bmp2a is mainly expressed in peripheral tissues (such as spleen, muscle, and heart). This could be because *bmp2a* genes and *bmp2b* genes play different roles in teleosts, and *bmp2b* may play an important role in the formation of IBs.

### 3.3. The Relationship between the Structure and Function of bmp2

Generally speaking, the structure of a protein determines its function [45]. In order to explore possible functional differences between the various subtypes of the *bmp2* gene family (*bmp2a* and *bmp2b*), we compared the amino acid sequences in the two proteins of multiple species. Through analysis of the important active sites of the bmp2a and bmp2b protein sequences of multiple species, we discovered that the bmp2a and bmp2b proteins in all species are conserved in amino acid around the 2HP ligand binding site, and the three-dimensional structures of bmp2a and bmp2b are also quite similar. It was also discovered that the spatial structure of zebrafish bmp2a and bmp2b contains a common receptor protein with a serine/threonine kinase activation site, and this indicates that bmp2a and bmp2b could have similar functions.

## 4. Methods and Materials

### 4.1. Acquisition of bmp2a and bmp2b for Nucleotide and Protein Sequences

A total of 37 different vertebrate species, containing nine mammals and 28 species of fish were observed for this study. Data were obtained using two different methods. For the first method, unreported *bmp2a* and *bmp2b* sequences from 20 species of fish, which were results from complete genome data produced by us and our collaborators (Table 1) was used. For the second method, published *bmp2a* and *bmp2b* sequences were downloaded from the public databases Ensembl and GenBank (Table S1). Potential homology-based *bmp2a* and *bmp2b* genes were recovered in detail from fish genomes through the use of tBLASTn [46] with an e-value of 10^−5^. The BLAST results were then treated by Perl script in order to find the best hit for each alignment. Finally, GeneWise v2.2.0 [47] was employed in order to predict the *bmp2a* and *bmp2b* genes from the best hits.

### 4.2. Phylogenetic Analysis and Sequence Alignment

For further phylogenetic analysis, we utilized protein and nucleotide protein sequences of all collected *bmp2a* and *bmp2b* genes. To summarize, MAFFT software [48] was used in order to align protein sequences of bmp2 and a maximum likelihood (ML) phylogenetic analysis was conducted, using the RAxML8.0.17 [49,50]. In addition, ML phylogenetic trees of the bmp2a and bmp2b isotypes were created, using their corresponding coding sequences by FastTree v2.1.7 [51]. We also downloaded a protein model of human bmp2 from the public Protein Data Bank (PDB) in order to compare structural differences among the fish bmp2a and bmp2b.

### 4.3. Analyses of Conserved Synteny and Gene Structures

In order to assess the conservation of *bmp2a* and *bmp2b* genes, we observed different genes, which are present in the downstream and upstream regions of each *bmp2a* and *bmp2b* paralog. Associated genomic data was also received from GenBank and our lab, as mentioned above. The zebrafish genome was utilized as the reference standard for the discovery of any *bmp2a* and *bmp2b* downstream and upstream regions. The genome assemblies of a variety of species of fish were investigated using the BLAST software, and the best hit was chosen, using a Perl script. GeneWise v2.2.0 was applied in order to forecast *bmp2a* and *bmp2b* gene structures.

### 4.4. qRT-PCR Analysis of bmp2a and bmp2b for Nile Tilapia and Bluntnose Black Bream

In order to discover the distribution of tissues of *bmp2* genes, we chose two representative fish. The Nile tilapia with fewer IBs and the bluntnose black bream with more IBs were selected. Total RNA was isolated from rib, IB, heart, spleen, liver, brain, and fin of the adult bluntnose black bream and Nile tilapia. With the use of an innovative method, the total RNA of IBs, ribs, and fins was isolated (Chinese Patent No.201310673534) for bone-related tissues. After using the PrimeScript^®^ RT reagent kit, following DNase treatment, 1 μg total RNA was reverse-transcribed to single-strand cDNA (Takara, Dalian). The transcriptional levels of *bmp2a* and *bmp2b* in various adult tissues were measured using a quantitative real-time PCR (qRT-PCR) assay in a Roche LightCycler 480 (Roche, USA). qRT-PCR was conducted using an AceQ^®^ qPCR SYBR^®^ Green Master Mix (Vazyme, Nanjing, China). The β-actin was used as a control. PCR primers (Table S2) were utilized in order to intensify the blunt snout bream *bmp2a*, *bmp2b,* and the β-actin. PCR reactions were performed for each reaction well containing 20 μL PCR mixture, composed of 1 μL of each specific primer, 1 μL cDNA, 10 μL Green Master Mix (Table S2), and 7 μL ddH_2_O. The reaction mixture was initially incubated at 95 °C for 3 min, followed by 40 cycles of 72 °C for 20 s, 60 °C for 30 s, and 95 °C for 15 s, and, finally at 4 °C for 5 min. The comparative expression ratio was normalized for the target genes with an internal reference β-actin gene and expression levels of *bmp2a* and *bmp2b*. Final results were determined using the 2^−ΔΔCt^ method [52].

### 4.5. Tertiary Structure and Function Prediction of Zebrafish bmp2 Protein

As mentioned earlier [53], I-TASSER was used to forecast the tertiary functions and structures of bmp2a and bmp2b. Through C-score, the confidence of models is measured quantitatively, based on the importance of the convergence parameters of the structure assembly simulations and threading template alignments. Ordinarily, a C-score is in a range where a high value supports the corresponding model with high confidence.

## 5. Conclusions

On the whole, we gained new insights into the fish *bmp2* gene family by comparing genomics and transcriptional studies. We acknowledged the presence of *bmp2a* and *bmp2b* in fish and noted changes in gene copy numbers and the distribution of tissue between the species. We predicted that the spatial structure of bmp2a and bmp2b in zebrafish would contain a common receptor protein with a serine/threonine kinase activation site, indicating that the *bmp2a* and *bmp2b* genes have high homology, which also implies that the site is likely to be a part of maintaining the function of the *bmp2* gene family in zebrafish. Our results also provide strong evidence to support the findings that *bmp2b* is expressed primarily in fishbone-related tissues (such as IB, fins, and ribs) and *bmp2a* is expressed mostly in peripheral tissues (such as spleen, muscle, and heart), while *bmp2b* may play a significant role in the formation of IBs in teleosts.

## Figures and Tables

**Table 1:** The copy number of *bmp2* genes from the fishes sequenced by our lab or collaborators.

Class	Common Name	Species Name	*bmp2a*	*bmp2b*
Actinopterygii	Snakehead fish	*Channa argus*	0	1
	Large yellow croaker	*Larimichthys crocea*	0	1
	Giant-fin mudskipper	*Periophthalmus magnuspinnatus*	0	1
	Black porgy	*Acanthopagrus schlegelii*	0	1
	Cave-restricted golden-line fish (Sa)	Sinocyclocheilus anshuiensis	2	2
	Semi-cave-dwelling golden-line fish (Sr)	Sinocyclocheilus rhinocerous	2	2
	Surface-dwelling golden-line fish (Sg)	Sinocyclocheilus grahami	2	1
	Kanglang fish	*Anabarilius sgrahami*	1	1
	Catfish	*Silurus asotus*	1	1
	Channel catfish	*ictalurus punctatus*	1	1
	Lined_seahorse	*Hippocampus erectus*	0	1
	Longjaw grenadier anchovy	*Coilia macrognathos*	1	0
	Spotted gar	*Lepisosteus oculatus*	0	1
	Golden arowana	*Scleropages formosus*	0	1
	Bluntnose black bream	*Megalobrama amblycephala*	1	1
	Atlantic herring	*Clupea harengus*	1	1
	Hilsa shad	*Tenualosa ilisha*	2	1
	Mexican tetra	*mexicanus*	1	1
	Zebrafish	*Danio rerio*	1	1
	Japanese medaka	*Oryzias latipes*	1	1
	Nile tilapia	*Oreochromis niloticus*	1	1
	Gilthead seabream	*Sparus aurata*	1	1
	Tongue sole	*Cynoglossus semilaevis*	1	1
	Japanese flounder	*Paralichthys olivaceus*	1	1
	Atlantic salmo	*Salmo salar*	2	2
	Fugu rubripes	*Takifugu rubripes*	1	1
Sarcopterygii	Coelacanth	*Latimeria chalumnae*	0	1
Holocephali	Australian ghostshark	*Callorhinchus milii*	1	1
Mammalis	Platypus	*Ornithorhynchus anatinus*	1	1

## Supplementary Materials

The following are available online: https://www.mdpi.com/1422-0067/20/24/6137/s1. Table S1, the accession numbers for bmp2 sequences and Table S2, primers used in the present study.

## Abbreviations

TGF-β	Transforming growth factor beta
LAP	Latency associated peptide
MAFFT	Multiple sequence alignment based on fast Fourier transform
hao1	Hydroxyacid oxidase 1
tmx4	Thioredoxin related transmembrane protein 4
Napba	*N*-ethylmaleimide-sensitive factor attachment
crls1	Cardiolipin synthase 1
mcm8	Minichromosome maintenance 8
Gpcpd1	Glycerophosphocholine phosphodiesterase 1
trmt6	tRNA methyltransferase 6
fermt1	Fermitin family member 1
TAAR12j	Trace amine associated receptor 12j
TAAR12e	Trace amine associated receptor 12e
TAAR12g	Trace amine associated receptor 12g
TAAR12f	Trace amine associated receptor 12f
TAAR12a	Trace amine associated receptor 12a
TAAR12b	Trace amine associated receptor 12b
TAAR12c	Trace amine associated receptor 12c
TAAR10	Trace amine-associated receptor 10
