# Supplementary material for: A Comparative Genomic and Transcriptional Survey Providing Novel Insights into Bone Morphogenetic Protein 2 (bmp2) in Fishes
Source: Int J Mol Sci. 2019 Dec 5;20(24):6137. doi: 10.3390/ijms20246137 (PMC6940749; doi:10.3390/ijms20246137)
Supplement: Supplementary file 1 [file ijms-20-06137-s001.zip › Supplementary Files/Table S1.docx]

**Table S1: The accession numbers for known bmp2 sequences**

| Class | Common Name | Species Name | bmp2 | Nucleotide Sequence | Protein Sequence |
| --- | --- | --- | --- | --- | --- |
| Mammalis | Human | *Homo sapiens* | a | NM_001200.4 | NP_001191.1 |
|  | olive baboon | *Papio anubis* | a | XM_021921220.1 | XP_021776912.1 |
|  | chimpanzee | *Pan troglodytes* | a | XM_514508.3 | XP_514508.2 |
|  | house mouse | *Mus musculus* | a | NM_007553.3 | NP_031579.2 |
|  | cattle | *Bos taurus* | a | NM_001099141.1 | NP_001092611.1 |
|  | ass | *Equus asinus* | a | XM_014828745.1 | XP_014684231.1 |
|  | domestic cat | *Felis catus* | a | XM_003983769.5 | XP_003983818.1 |
|  | rabbit | *Oryctolagus cuniculus* | a | NM_001082650.1 | NP_001076119.1 |
| Actinopterygii | zebrafish | *Danio rerio* | a | KM820423.1 | AJF23293.1 |
|  | zebrafish | *Danio rerio* | b | NM_131360.2 | NP_571435.1 |
|  | Japanese medaka | *Oryzias latipes* | b | NM_001104908.1 | NP_001098378.1 |
|  | Nile tilapia | *Oreochromis niloticus* | b | XM_019346038.2 | XP_019201583.1 |
|  | gilthead seabream | *Sparus aurata* | b | JF261172.1 | ADZ46051.1 |
|  | tongue sole | *Cynoglossus semilaevis* | b | NM_001328245.1 | NP_001315174.1 |
|  | Japanese flounder | *Paralichthys olivaceus* | b | XM_020091631.1 | XP_019947190.1 |
|  | Atlantic salmo | *Salmo salar* | b | NM_001173834.1 | NP_001167305.1 |
|  | Fugu rubripes | *Takifugu rubripes* | b | XM_003971524.2 | XP_003971573.1 |
