# Supplementary material for: A Comparative Genomic and Transcriptional Survey Providing Novel Insights into Bone Morphogenetic Protein 2 (bmp2) in Fishes
Source: Int J Mol Sci. 2019 Dec 5;20(24):6137. doi: 10.3390/ijms20246137 (PMC6940749; doi:10.3390/ijms20246137)
Supplement: Supplementary file 1 [file ijms-20-06137-s001.zip › Supplementary Files/Table S2.docx]

**Table S2: Primers used in the present study**

| Primers | Sequences (5' - 3') | Purpose |
| --- | --- | --- |
| *O.niloticus*-bmp2a-F | CGACATTGCTTTTGAGTACCCAG | qRT-PCR |
| *O.niloticus*-bmp2a-R | ATCAATCTGATGACGGTAGAGCC |  |
| *O.niloticus*-bmp2b-F | GACCTTTACCGTATGCATTCAGC | qRT-PCR |
| *O.niloticus*-bmp2b-R | ACCTGATCCCTGTAAATGCGTAG |  |
| *O.niloticus*-β-actin-F | TGGTGGGTATGGGTCAGAAAG | qRT-PCR |
| *O.niloticus*-β-actin-R | CTGTTGGCTTTGGGGTTCA |  |
| *M.amblycephala*-bmp2a-F | TCCTCTCTGTCTCAGGATGTCTT | qRT-PCR |
| *M.amblycephala*-bmp2a-R | TGGAAGCTCCTGATAGTATTCGC |  |
| *M.amblycephala*-bmp2b-F | GCCTGAGAGGAAAAACAACACAG | qRT-PCR |
| *M.amblycephala*-bmp2b-R | AAGTCTGGTAAGAGGCTCTTTGG |  |
| *M.amblycephala*-β-actin-R | AGGTCATCACCATTGGCAAT | qRT-PCR |
| *M.amblycephala*-β-actin-R | GATGTCGACGTCACACTTCAT |  |
